# Bacteriological Assessment of Bottled Drinking Water Available at Major Transit Places in Mangalore City of South India

**DOI:** 10.1155/2018/7472097

**Published:** 2018-10-25

**Authors:** Nitin Joseph, Sevitha Bhat, Subhani Mahapatra, Ayush Singh, Sajal Jain, Ahamed Unissa, Namritha Janardhanan

**Affiliations:** ^1^Post Graduate Diploma in Family Medicine, Associate Professor, Department of Community Medicine, Kasturba Medical College, Light House Hill Road, Manipal Academy of Higher Education, Mangalore, India; ^2^Associate Professor, Department of Microbiology, Kasturba Medical College, Light House Hill Road, Manipal Academy of Higher Education, Mangalore, India; ^3^MBBS Student, Kasturba Medical College, Light House Hill Road, Manipal Academy of Higher Education, Mangalore, India

## Abstract

**Introduction:**

Safe drinking water is essential for human life. It is generally considered that bottled water is safe for usage by people. For long-distance travelers, it serves as the only source of reliable drinking water. But, several studies have reported that bottled water does not always meet the acceptability standards.

**Objectives:**

To assess the bacteriological and physical quality of bottled water marketed in major transit areas and to check its compliance with national standards.

**Methods:**

The investigating team visited retail shops at three main transit sites for long-distance travelers in Mangalore city. A total of 24 water bottles of 12 brands were randomly selected. The analysis of total viable count (TVC) was done to assess the bacteriological quality of samples.

**Results:**

In 3(12.5%) samples, all of which were of local brands, batch number, the period of manufacture, and the period of expiry were not mentioned. Odor and floating bodies were present in one sample each. Five (20.8%) water bottles had been enriched with minerals. Ozone treatment was the most commonly 22(91.7%) used method for disinfection of water. In only 15(62.5%) samples, the bacterial contamination was within acceptable limits certified for drinking purposes. Water samples manufactured by multinational companies (*p*=0.018), those with batch number mentioned (*p*=0.042), the best period of manufacture (*p*=0.036), and long expiry dates (*p*=0.028) were acceptable for usage.

**Conclusion:**

Surveillance of bottled water manufacturing industries in the settings on a regular basis needs to be done by regulatory agencies. These measures will ensure safe and wholesome bottled water for public usage.

## 1. Introduction

Bottled water is generally regarded safe for usage by people. It serves as the only reliable source of drinking water accessible for long-distance travelers. Its usage rate in parts of Asia is estimated to be around 27% [1]. With respect to bottled water consumption, India is rated among the top ten countries in the world. Bottled water production companies are one of the fastest growing industrial sectors in this part of the world. Presently, there are more than 3400 bottling plants in India. Half of these are concentrated in the southern regions of India [2].

Demand for bottled water has resulted in springing up of several small-scale entrepreneurs involved in its production and distribution. However, with increasing demand, serious concerns about its quality and safety have arisen subsequently. The chemical and microbiological qualities of packaged water of some manufacturers have been found to be in violation of national standards [3]. Studies done in India and other parts of the world have reported that bottled water was contaminated with harmful disease-causing microorganisms at various stages of its production [4–6]. Consumption of contaminated water in India has led to frequent outbreaks of waterborne diseases such as cholera, typhoid, and hepatitis A and E [7].

The manufacturing plants of most companies of bottled water in India are situated in unhygienic locations like agricultural fields or estates. Most companies use bore wells as source of water. Here, water is pumped out from depths varying from 80 to 500 feet below the ground [8, 9]. The less likely sources of packaged water are from public drinking water systems such as Municipality supply water [10].

Ground water has quality problems due to salinity (particularly in coastal areas) and contaminants like agrochemicals, nitrates, fluoride, iron, and arsenic [11, 12]. The ground water available in about one-third of India's districts was found to be unfit for drinking. This was because of the presence of contaminants exceeding the tolerance levels [12].

Significant levels of pesticides like organochloride compounds (lindane, DDT, and endosulfan) and organophosphorus compounds (malathion and chlorpyrifos) have been reported in fresh water systems and in the bottled water samples collected from some major cities in India [8, 9]. These observations imply that the technology currently being used for treating raw water is insufficient to have safe water for consumption [9].

Hence, periodic surveillance of packaged drinking water like bottled water is very much essential. This will serve the dual purpose of monitoring the standards of bottled water production industries as well as help in giving reassurance of quality to users.

This study was therefore done to assess the bacteriological and physical quality of bottled water marketed in major transit areas within a city and check its compliance with national standards.

## 2. Materials and Methods

This cross-sectional study was done in April 2016 in Mangalore city of Karnataka state situated in south India. The ethical approval was obtained from the institutional ethics committee.

It was conducted at three main transit sites for long-distance travelers in Mangalore namely Karnataka State Road Transport Corporation bus stand, Mangalore central railway station, and Mangalore junction railway station.

The sample size was calculated using the formula *Z*_*α*_^2^*pq*/*d*^2^. Based on the findings of a previous study, which reported 94% of the bottled water samples within acceptable standard for usage [13], and at 95% confidence intervals and 90% power, the sample size of a total of 24 bottled water was calculated.

Two bottles each of twelve different brands available at various retail outlets at these sites were randomly purchased using a simple random sampling method.

The selected bottles before purchase were inspected for good condition of the cap and the protective seal. The brand name, dates of manufacture and expiry, batch number, Indian Standard Items (ISI) symbol, mineral contents, the process of water purification employed, and place of manufacture were documented in a pro forma.

It is mandatory for all the manufacturers of bottled water to obtain the ISI certification from Bureau of Indian Standards (BIS) as per the Government notification issued in the year 2000 by Ministry of Health and Family Welfare [9]. The BIS staff does a check of the water samples from these plants in an independent laboratory. Only if the samples are reported safe for consumption, official confirmation and license number are given by them to the plant for commencing commercial production [14]. They also assess the infrastructure facilities of the manufacturing plants by surprise inspections and periodic testing of samples available at the manufacturing and marketing sites [9]. The BIS standard for bottled drinking water follows IS 14543 : 1998 guidelines covered under the relevant Prevention of Food Adulteration Act of the Government [9, 10].

The type of physical, chemical, and microbiological tests to be done for the water samples to be tested is prescribed under IS: 3025 guidelines [14]. The various treatment procedures done for packaged drinking water at the factory constitute decantation, filtration by sand, carbon and micron cartridge filter (to remove suspended and colloidal impurities), filtration with ultramembrane filter (to remove fine suspended solids, protozoa, bacteria, and viruses), depth filter, cartridge filter, activated carbon filtration (to remove organic impurities), ozonization (to eliminate bacteria), ultraviolet treatment (to inactivate bacteria), silver ionization, ion exchange and reverse osmosis (to remove dissolved solids, heavy metals, fluoride, and pesticides/fertilizer residues), and procedures like demineralization and remineralization to meet the prescribed standard of the packed item [8, 10]. Among the chemical disinfectants, free chlorine is most commonly used to treat water [9]. Water is then filled in cleaned and rinsed containers. Containers are visually inspected for any suspended matter and for leakage against an illuminated screen [10]. The manufacturer needs to do periodic in-house testing of packaged water as stated in the BIS document. The various tests done at their quality control laboratories include examination of total dissolved solids, turbidity, pH, color, and conductivity in addition to routine bacteriological analysis [9]. The standard also prescribes that the shelf life of the product shall be declared and marked on the product by the manufacturer based on their in-house studies [10]. The source water should also be tested once in three months for physical, chemical, and bacteriological parameters by the manufacturers and the records to be maintained by them [10].

However, in spite of all these protective measures, presence of contaminants in bottled water implies that the treatment process at the plants is not effective [8]. Hence, it was necessary to check the quality of packaged water available at this setting.

The plastic bottles that were purchased from various outlets for analysis in this study had a capacity of 500–1000 ml. All the bottles were transported to the laboratory of the Department of Microbiology of this institution. Each bottle was vigorously shaken and observed for turbidity, odor, and floating bodies. The analysis of total viable count (TVC) was done using the standard plate count method. TVCs are good indicators of general contamination and of the overall quality of production of the product [15–17]. It gives a quantitative estimate of the concentration of microorganisms in a water sample to be tested. The count represents the number of colony-forming units (CFUs) per ml of the sample. The reported count is the number of colonies counted multiplied by the dilution used for the counted plate. A high TVC count indicates a high concentration of microorganisms, which may indicate poor quality for drinking water or foodstuff [18].

The microbiological test was done within 2 hours of purchase of the bottles from the point of sale [19]. The determination of total heterotrophic bacteria was done using serial dilution and pour plate technique. For this, tenfold serial dilutions in sterile water were carried out for each water sample brought for testing. One ml from the 10^th^ test tube was aseptically taken on two occasions and placed in two different sterile 4-inch diameter Petri dishes. Then, 20 ml of molten plate count agar cooled to 50°C was added to each plate and mixed thoroughly. The mixtures were allowed to solidify. One plate was incubated at 22°C and the other at 37°C for 48 hours. After incubation, the number of bacterial colonies in both the plates was counted, and the average was reported as CFUs per milliliter of the tested sample [20].

The recommendations of BIS for acceptability of packaged natural mineral water was used for comparison of physical and bacterial quality [10]. According to this, the viable count limit should not exceed 100 CFU per ml of the sample at room temperature. It should also be devoid of any turbidity, odor, or floating bodies.

Data were entered in Microsoft windows excel and were exported to SPSS version 16.0 (SPSS Inc., Chicago, IL, USA) for analysis.

Chi-square test, Fisher's exact test, and binary logistic regression analysis were done to test the association between variables with the status of acceptability of water samples. All statistical significance was assessed at the 5% level.

## 3. Results

Out of the total, 20 bottles were collected from the central bus stand area and two each from the two railway stations situated within the city limits. All these collected samples were found to have a sealed cap and were labeled with ISI certification.

Majority of the bottles (10; 41.7%) were manufactured by multinational companies. In 3 (12.5%) bottles, batch number, period of manufacture, and period of expiry were not mentioned. (Table 1). These were of brands manufactured by local (regional) companies. There was also a significant association between non mention of these parameters with bottled water manufactured by local companies using Fisher's exact test (*p*=0.0277).

Most common methods used for purification of water was by ozone treatment 22 (91.7%) followed by ultraviolet irradiation 19 (79.2%) and with reverse osmosis technique 16 (66.7%). (Table 2). In 16 (66.7%) samples, more than one purification technique was used by the manufacturers.

**Figure 1:**
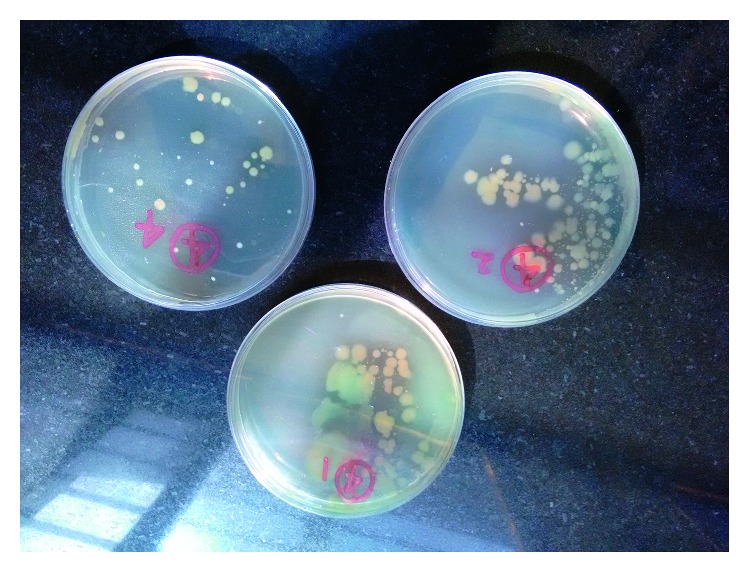
Determination of colony-forming units of total heterotrophic bacteria using serial dilution and pour plate technique.

There was no sample with turbidity. One sample was found to have odor while another contained floating bodies. The number of CFU per ml was <100 in 15 samples, 101–200 in 3 samples, 201–400 in 4 samples, and 401–500 in 2 samples. The mean CFU count was 123.4 ± 161.9, and the median count was 30 with the interquartile range (4, 275). The CFU count ranged from 1 to 500 (Figure 1). Therefore, in only 15 (62.5%) samples, the bacterial growth was within acceptable limits certified by BIS for drinking purposes. The nine unacceptable samples belonged to six brands, two each of three brands and one each of three different brands.

Five (20.8%) water bottles had been enriched with minerals. Three of these bottles were manufactured by multinational companies and two by local companies. The minerals comprised of magnesium sulphate in all 5 bottles, potassium carbonate in 3 bottles, and sodium chloride in two bottles.

Water samples manufactured by multinational companies (*p*=0.018), samples from bottles with the batch number mentioned (*p*=0.042), the best period of manufacture in the current month (*p*=0.036), and expiry date beyond six months (*p*=0.028) were found to be significantly acceptable for drinking purposes. (Table 3).

Multivariable analysis showed no association of any of the factors with acceptability of bottled water analyzed in this study. For the calculation of odds ratio for the characteristics introduced in this model, water bottles manufactured by regional companies, bottles manufactured in the previous month or without mention of manufacture date, and bottles due for expiry within the next 6 months or without mention of expiry date were taken as the reference (Table 4).

## 4. Discussion

Provision of safe drinking water is one of the most essential amenities to be made available for citizens in the modern world. Particularly, for long-distance travelers who need to be extra careful of their health but do not have other options, depend on these packaged water sources. Therefore, it is a matter of concern that only about two-thirds of the bottled water tested was suitable for drinking in the present study. This was similar to the findings of another study done in Mangalore in 2002 which reported 66.7% of the sampled bottled water safe for consumption [21]. This meant that the situation of hygienic status of bottled water available in this city has not shown any improvement with time. It could be because of the reason that, this issue was not given priority as much as other public health issues concerning this city. In other studies done in India, the acceptability of bottled water ranged from 60% [4, 22, 23], 83% [24], 90% [25], and even 100% [26–28]. In studies done in other parts of Asia, the acceptability of bottled mineral water samples ranged from 50% [29], 64.2% [19], 97.1% [30] and a study done in Iran even reporting 100% [31]. In studies done in Africa, it was 67.4% [20], 70% [32], 71.4% [17], 75% [33], 85% [16], 88.9% [34], 90% [35], 94% [13] and a study done in Uganda [36] and Nigeria [37] reporting 100%.

In a study done in Pakistan, the TVC in CFU/ml was <1 in 40%, 15–20 in 24%, 20–200 in 10%, 200–300 in 13%, and >300 in 13% [19]. In a study done in different parts of North India, around 2% of the samples tested had bacterial counts of more than 1000 CFU/ml [24]. The contamination level of water samples reported in these above-mentioned studies was therefore much more than our observations. However, another study done in Chennai, India, reported that bacterial counts ranged from 0 to 41 CFU/ml among all the water samples tested, which was much lesser than that observed in the present study [28]. The presence of heterotrophic bacteria in the bottled water causes significant health risk particularly for children, elderly, and immunocompromised individuals [38]. Its presence in bottled water is also an indicator of poor practices involved in the manufacturing processes.

The kind of bacteria found in the bottled water has previously been reported to have multiple drug resistance in samples collected from different parts of India [23]. Safety of bottled drinking water can be ensured with sealed caps on bottles, hygienic filling systems, the minimal time between production and sale, and use of nonreturnable plastic containers [4, 6, 34]. It was observed in a Nigerian study that contamination of packaged water aggravates as the product moves down the distribution chain [39]. Assessment of water quality is therefore required not only at various stages of production but also in postproduction stage [28]. This will ensure improvement in transportation and storage practices in the supply chain. Government and other stakeholders need to intensify surveillance activities of water treatment processes at packaged water industries. This will ensure that strict hygienic measures are followed, resulting in safe and quality bottled water being available at various retail outlets for public use.

Among the physical parameters, turbidity was absent in all the water samples tested in this study. This was comparable to the observations of a study done in Ghana [16]. Turbidity of water depends on the amount of particulate matter present in it. This interferes with the disinfection process of water [40]. It also affects the taste, odor, and the color of the water [41].

Odor and floating bodies were present in one sample each in this study, which again is an indicator of poor manufacturing and storage practices. In a study done in Nigeria, total suspended solids (particulate matter) was absent in all bottled water samples under investigation [33]. Similarly, in another study done in Telangana, India, all the bottled water samples were colorless and had no objectionable odor and taste [26].

The batch number, period of manufacture, and period of expiry were not mentioned on three bottles, all of which were manufactured by local companies. A Nigerian study also reported that none of the bottled water brands had mentioned the batch number [33]. Batch number is very essential for any manufactured product. In the event of discovery of any abnormality, with the help of the batch number, the entire lot the product can be identified and recalled from the market by the company [33].

In this study, five water bottles had been enriched with minerals and were labeled with these specifications. The mineral composition was not stated in any of the sampled water bottles in the Nigerian study. However, all the bottled water samples in the latter study had mentioned manufacturing and expiry dates, unlike our observations [33]. All the samples without batch number, manufacture date, and expiry date were found unacceptable for drinking in the present study. Therefore, public enlightenment on particulars which they need to look out for on the package label before purchasing bottled water is essential. The local companies that manufacture products without complete label need to be questioned on these issues.

Moreover, three-fourth samples of locally manufactured bottled water were found unfit for consumption in this study. Springing up of several small-scale entrepreneurs engaging in the production of mineral water, without due regard to hygienic practices, has resulted in Mangalore. This might be due to the high demand of water as a consequence of the hot and humid weather seen mostly at this place. Packaged water manufactured by these regional companies may lack the guarantee to meet the set standards for drinking water quality. Therefore, identification of all local companies involved in its production, licensing, and renewal of licensing of these companies, by concerned authorities, is required in order to safeguard the health of the consumers [27].

On the other hand, bottled water manufactured by multinational companies was mostly of acceptable standards, as observed in this study. These companies have better infrastructure and a wide variety of sophisticated equipment for quality production of items. The production processes by these companies are done by qualified personnel who are closely supervised by trained professionals. The licensing of these companies is also periodically renewed [20].

## 5. Conclusion

More than one-third of bottled water available at major transit sites in Mangalore was found to be not suitable for usage. The absence of essential labeling items like batch number, date of manufacture and expiry on the containers, and unacceptability of water for drinking was seen significantly among local brands of bottled water. This infers toward noncompliance with stipulated guidelines in the production process by local manufacturers. Therefore, surveillance of packaged water manufacturing industries by regulatory agencies at this setting needs to be stepped up. ISI certification authorities also need to do a random sample check of all their licensee products. Imposition of sanctions should also be done on defaulting industries to ensure effective compliance with BIS standards. The distributors, retailers, and consumers also need to be made aware of the identification, reporting, and removal of problematic bottled water available at points of sale. These measures will ensure that safe and wholesome bottled water is available for public usage.

## Figures and Tables

**Table 1:** Characteristics of the packaged drinking water bottles.

Characteristics	Number	Percentage
Manufacturers		
Local companies	8	33.3
National companies	6	25.0
Multinational companies	10	41.7
Period of manufacture (*n*=21)		
Current month	14	66.7
Previous month	7	33.3
Period of expiry (*n*=21)		
Within the next 6 months	13	61.9
Beyond 6 months	8	38.1
Site of collection		
Bus stand	20	83.3
Railway station	4	16.7
Total	24	100.0

**Table 2:** Purification techniques used for packaged drinking water bottles (*n*=24).

Purification techniques	Number	Percentages
Ozone treatment	22	91.7
UV irradiation	19	79.2
Reverse osmosis	16	66.7
Sand filtration	7	29.2
Activated carbon filtration	6	25.0
Multistage filtration	2	8.3
Micron filtration	2	8.3

**Table 3:** Association between various characteristics with bacteriological acceptability status of water samples.

Characteristics	Acceptable (%)	Not acceptable (%)	Total
Manufacturers			
Regional companies	2 (25)	6 (75)	8
National companies	4 (66.7)	2 (33.3)	6
Multinational companies	9 (90)	1 (10)	10
			*X* ^2^ = 8.071, *p*=0.018
Site of collection			
Bus stand	11 (55)	9 (45)	20
Railway station	4 (100)	0 (0)	4
			*p*=0.259
Batch number			
Mentioned	15 (71.4)	6 (28.6)	21
Not mentioned	0 (0)	3 (100)	3
			*p*=0.042
Addition of minerals			
Yes	5 (100)	0 (0)	5
No	10 (52.6)	9 (47.4)	19
			*p*=0.118
Period of manufacture			
Current month	11 (78.6)	3 (21.4)	14
Previous month	4 (57.1)	3 (42.9)	7
Not mentioned	0 (0)	3 (100)	3
			*X* ^2^ = 6.629, *p*=0.036
Period of expiry			
Beyond 6 months	7 (87.5)	1 (12.5)	8
Within the next 6 months	8 (61.5)	5 (38.5)	13
Not mentioned	0 (0)	3 (100)	3
			*X* ^2^ = 7.138, *p*=0.028
No. of purification techniques used			
One	5 (100)	0 (0)	5
2 or 3	5 (45.5)	6 (54.5)	11
>3	5 (62.5)	3 (37.5)	8
			*X* ^2^ = 4.364, *p*=0.113
Total	15	9	24

**Table 4:** Multivariable analysis of characteristics influencing acceptability of water samples (*n* = 24).

Characteristics	Unadjusted OR	95% CI of unadjusted OR		*p* value	Adjusted OR	95% CI of adjusted OR		*p* value
		Lower	Upper			Lower	Upper	
Manufacturers	13.0	1.701	99.375	0.018	3.81	0.912	15.918	0.067
Manufacture date	5.5	0.912	33.184	0.036	2.908	0.503	16.8	0.233
Expiry date	7.0	0.174	4.811	0.028	0.968	0.172	5.441	0.971

## Data Availability

The data used to support the findings of this study are available from the corresponding author upon request.
